# Assessment of Bovine Herpesvirus Type 1 (BoHV-1) Stability and Infectivity on Copper, Zinc, and Stainless Steel Surfaces

**DOI:** 10.3390/vetsci13040381

**Published:** 2026-04-15

**Authors:** Dovilė Grigauskaitė, Raimundas Lelešius, Dainius Zienius, Raimundas Mockeliūnas, Algirdas Šalomskas

**Affiliations:** 1 Department of Veterinary Pathobiology, Veterinary Academy, Lithuanian University of Health Sciences, Tilzes 18, 47181 Kaunas, Lithuania; raimundas.lelesius@lsmu.lt (R.L.); dainius.zienius@lsmu.lt (D.Z.); algirdas.salomskas@lsmu.lt (A.Š.); 2 Institute of Microbiology and Virology, Veterinary Academy, Lithuanian University of Health Sciences, Tilzes 18, 47181 Kaunas, Lithuania; raimundas.mockeliunas@lsmu.lt

**Keywords:** bovine herpesvirus type 1, copper, zinc, stainless steel

## Abstract

Despite increased interest in virus survival on surfaces, data on bovine herpesvirus type 1 (BoHV-1) interactions with metal surfaces remain limited. This study aimed to assess the effects of copper, zinc, and iron on BoHV-1 viability, viral titre, and DNA stability under different conditions. MDBK-adapted BoHV-1 was used to investigate the virucidal effect of copper, zinc and stainless steel surfaces. The virus was exposed for 1 and 24 h under both wet and dry conditions. Inactivation was assessed based on changes in TCID_50_ log_10_ values, qPCR Ct results, and calculating half-lives of the virus and its DNA. Virus stability varied depending on surface type, environmental conditions, and duration of exposure. Copper demonstrated the strongest virucidal effect, significantly reducing viral titres and DNA levels under all conditions. After 1 h in wet conditions, copper reduced viral titre to 4.7 log_10_, while zinc and stainless steel showed minimal impact. Under dry conditions, copper reduced viral titres to the limit of detection after 24 h. Half-life analysis confirmed rapid inactivation on copper, with the shortest persistence observed across all conditions. Zinc showed moderate virucidal activity but required longer exposure times. These findings highlight copper’s superior antiviral properties and suggest its potential application in reducing viral transmission on surfaces.

## Simple Summary

Certain metals are known to affect infectious pathogens, yet their effects on virus survival on surfaces remain unclear. While this has been widely studied for human viruses, less is known about animal viruses such as bovine herpesvirus type 1 (BoHV-1). In this study, we examined how long BoHV-1 survives on different metal surfaces, including copper, zinc, and stainless steel, under both wet and dry conditions over different periods of time. Copper was highly effective at inactivating the virus under both wet and dry conditions, whereas zinc showed a moderate effect and stainless steel had little impact. These findings highlight the potential of copper surfaces as a practical way to inactivate the virus, helping to limit virus spread and reduce the risk of infections or outbreaks.

## 1. Introduction

For centuries, it has been known that certain metals, due to specific biophysical and biochemical properties, can affect the pathogens and/or their biological environment. However, the mechanisms of these properties are not yet fully understood, and it is believed that there may be many causal relationships behind this effect. In the context of global outbreaks of infectious diseases, the applied science market has seen the emergence of specialized virological studies aimed at investigating the influence of different surfaces on the resilience of viruses [1,2,3]. Both enveloped and non-enveloped viruses, as well as DNA and RNA viruses, are the subjects of research due to their differing biological and structural properties. The majority of these studies, however, have focused on human viruses, including coronaviruses, herpesviruses, and lentiviruses which are responsible for diseases such as COVID-19, herpes simplex, and HIV infection, respectively, whereas the effects on animal viruses remain largely underexplored [1,2,4].

It is known that viruses of the same family or genus can exhibit both similar and differing sensitivities to various substances; therefore, only through experimental studies can the inactivation potential or rate of specific virus species be determined [5]. Among enveloped, DNA-containing viruses, human herpes simplex viruses (HSV), which belong to the *Orthoherpesviridae* family, have been studied most extensively, whereas bovine herpesviruses have received comparatively little attention.

Bovine herpesviruses are of high veterinary significance because they are associated with severe respiratory, reproductive, and systemic diseases in cattle, leading to economic losses due to reduced milk production, weight loss, abortions, and impaired fertility. In addition, these viruses can establish latent infections, making eradication challenging and increasing the risk of recurrent outbreaks. They can spread through direct contact or indirectly through contaminated environments and surfaces [6].

Four viruses that were formerly known as bovine herpesviruses are classified under the *Orthoherpesviridae* family, according to the International Committee on Taxonomy of Viruses (ICTV). The virus names have since been revised. Under the current ICTV taxonomy, the *Orthoherpesviridae* family is divided into three subfamilies: *Alphaherpesvirinae*, *Betaherpesvirinae*, and *Gammaherpesvirinae*. The *Alphaherpesvirinae* subfamily includes three bovine viruses of veterinary importance and one of them is from the genus *Varicellovirus*: *Varicellovirus bovinealpha*1 (BoHV-1). BoHV-1 is the most important virus in veterinary medicine, and therefore it was selected for this study [7].

Studies have shown that the stability of viruses varies significantly depending on both the structure of the virus [8,9,10,11] and the type of material surface, such as metals (copper, silver, zinc, stainless steel), glass, cotton, both paper and polymer banknotes, and others [10,12,13]. These observations have prompted researchers to explore the physical and chemical properties of various surface coatings that could reduce virus stability and, consequently, their potential transmission to healthy individuals [14].

Several key factors can influence the survival of viruses on surfaces. First, the characteristics of the contact material, such as surface type, porosity, and roughness. Second, physical and abiotic factors, including relative humidity, temperature, and exposure to light (different wavelengths of the solar spectrum). Third, biological factors, such as the viral structure or the presence of microbial biofilms. Fourth, chemical conditions, including pH, the presence of reactive ions, adsorption state, organic matter, or specific chemical components in the environment. Notably, a significant subpopulation of viruses can be stabilized and protected by surrounding organic material, such as saliva or mucus droplets, depending on the structure of their envelope. In these cases, external substances and structures—such as bacteria, lipids, proteins, or carbohydrates in the contact environment—may further enhance viral resistance [9,15].

In recent years, scientific and applied research has increasingly focused on the use of self-decontaminating surfaces to reduce the presence of pathogens in the air and on various surfaces. Various materials are being explored as additional safety measures to prevent disease transmission; for example, surface coatings containing zinc oxide (ZnO) and copper oxide (CuO) nanoparticles have demonstrated antimicrobial and antiviral properties [16,17].

Due to their low cytotoxicity index and high antimicrobial activity, Zn/ZnO nanoparticles (NPs) can also be used as disinfectants and have been incorporated into commercial products, for example, in food packaging components [16,18]. Copper is the most commonly used metal for the development of antimicrobial surfaces due to its effective contact denaturing effect [19]. It can be used pure, alloyed, in composites of different structures, and in NPs form in a wide range of products, including door handles, handrails, and textiles [20,21].

Various forms of copper compounds, especially CuO particles, are considered among the most popular in the field of antimicrobial research. These copper compounds have been widely used in industry, due to their versatile physical properties and cost-effectiveness in production [6,22]. It has been demonstrated in a number of research projects that CuO exerts an inhibitory effect on viruses, but most of the studies have focused on the effectiveness of Cu against bacterial infections [6,23]. Previous results from bioresistance studies have shown strong antiviral activity of ZnO against HSV type 1 (HSV-1) [24].

More than thirty metals can interact with microorganisms and have antiviral effects. This list includes gold, silver, copper, iron, lead, zinc, and numerous others [14]. However, there is little information regarding the interaction between animal viruses, especially BoHV-1, and various metal surfaces. The use of biocidal surfaces can help reduce the spread of viruses transmitted by contact with contaminated surfaces [25]. The disinfectant properties of certain metals, such as silver and copper, have been documented since antiquity [26]. Cattle farms use a lot of equipment made of metals, mainly steel or galvanized steel, and less commonly stainless steel. Copper or its alloys are also used on farms, for example, in parts for watering trough valves, valves for drinking water, and milk lines. Iron does exhibit antiviral activity, but viruses can persist for relatively long periods on its surface, which limits its practical application [12].

Therefore, the aim of the study was to assess the stability and infectivity of BoHV-1 on copper, zinc, and stainless steel surfaces under both wet and dry conditions.

## 2. Materials and Methods

### 2.1. Metal Surfaces

In this study, metal prototypes of copper, zinc and stainless steel, each measuring 10 × 10 mm (area 100 mm^2^, thickness 1 mm) and produced by the Department of Veterinary Pathobiology, Faculty of Veterinary Medicine, Lithuanian University of Health Sciences, were used. The technical specifications of the metal surfaces are provided in Table 1. Prior to use, all surfaces were stored in the dark at 20 °C and disinfected with 70% ethanol.

### 2.2. Cells and Virus

The study utilized the Madin–Darby bovine kidney (MDBK/NBL-1, Bov.90050801) cell line, and the MDBK-adapted BoHV-1 strain 4016 [30]. MDBK cell line and BoHV-1 strain 4016 were provided by Dr. I. Jacevičienė from the Department of Virus Research at the National Food and Veterinary Risk Assessment Institute in Lithuania.

The MDBK (NBL-1, CCL-22) cells were cultivated in Dulbecco’s Modified Eagle Medium and Nutrient Mixture F-12 (DMEM/F12, Gibco, Thermo Fisher Scientific, Paisley, UK) supplemented with 10% fetal bovine serum (FBS, Gibco, UK) in an incubator at 37 °C with 5% CO_2_. To prevent bacterial contamination, the medium was supplemented with penicillin (100 U/mL) and streptomycin (100 mg/mL) (Pen Strep, Gibco, Thermo Fisher Scientific, Waltham, MA, USA).

BoHV-1 was propagated in MDBK cells grown in T-25 flasks (TPP Techno Plastic Products AG, Trasadingen, Switzerland). After infecting the cells with the virus, DMEM/F12 maintenance medium supplemented with antibiotics and 2% FBS was added for further cultivation. After 48 h, the flasks containing the infected cells were examined under a microscope to assess cytopathic effect (CPE). Following examination, the flasks were frozen and thawed three times. The viral medium was clarified by centrifugation at 1000× *g* for 10 min, aliquoted into 1 mL tubes, and stored at −80 °C until use [31].

### 2.3. Cytotoxicity Control

The cytotoxicity of metal surfaces was investigated, as described by Bakhet et al. [32] in accordance with ISO 10993-5:2009. Briefly, the cytotoxicity of metal surfaces 10 × 10 mm was assessed in MDBK cells using the MTT assay. Cells were seeded at 1 × 10^4^ cells/well in a 96-well plate and grown at 37 °C for 24 h.

Metal surfaces were placed in separate wells of 6-well plates, and 10 μL of DMEM/F12 containing 5% FBS was added to each surface, forming a medium layer approximately 100 μm thick. The surfaces with the medium were then covered with cover slips and incubated at room temperature for 24 h. After incubation, extracts were diluted 1:25 to 1:1600 in medium containing 2% FBS and applied to cells. The cells were then microscopically observed and compared with control cells after incubation at 37 °C in a 5% CO_2_ incubator for 72 h. The MTT assay was then carried out. Absorbance was measured at 620 nm, and cell survival percentages were calculated [32].

### 2.4. Determination of Virucidal Effect

The virucidal effect of metal surfaces was assessed by calculating the log_10_ reduction in virus titre, as well as measuring changes in qPCR Ct values and the half-lives of viruses and viral DNA after 1 and 24 h of contact under both wet and dry conditions.

#### 2.4.1. Exposure of the Virus to Metal Surfaces

An evaluation of changes in viral titres after contact with surfaces was conducted in accordance with the guidelines established by the European Medicines Agency EMA [33].

For the metal surface study, BoHV-1 (7.7 ± 0.29 log_10_ TCID_50_ in dry conditions and 7.95 ± 0.25 log_10_ TCID_50_ in wet conditions) in DMEM/F12 maintenance medium supplemented with antibiotics and 2% FBS was used. The virus was thawed at 37 °C prior to the experiment. MDBK cells were also prepared, having been seeded into a 96-well titration plate two days before the study. The experiment was conducted in multiple stages, as illustrated in reactr.

All experiments were carried out under aseptic conditions in Class II biosafety cabinets to assess the effect of metal surfaces under both dry and wet conditions.

Each test material (10 × 10 mm) was placed into a separate well of a sterile 6-well plastic plate (TPP Techno Plastic Products AG, Trasadingen, Switzerland), and 10 µL of virus suspension was applied to the test surface using a dispenser.

**Figure 1:**
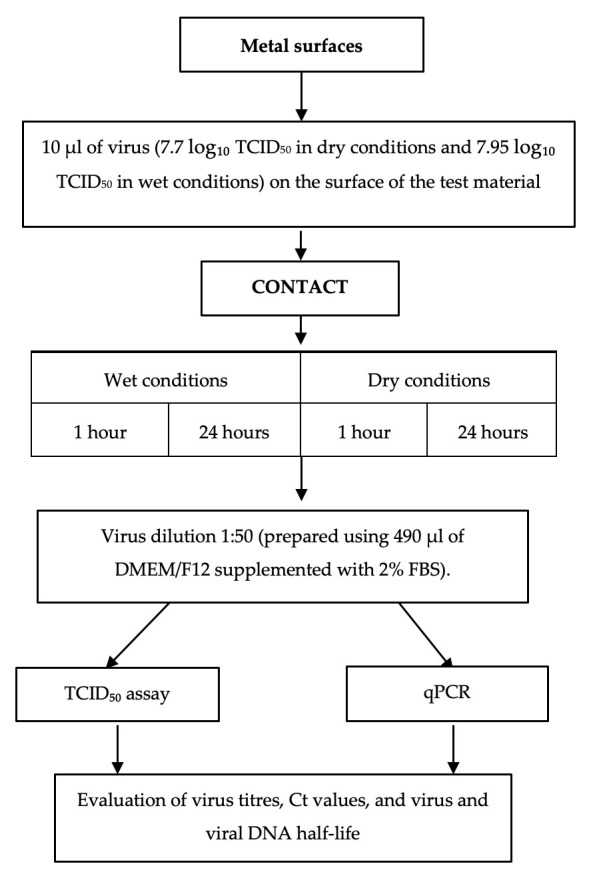
Testing scheme for the virucidal effect on metal surfaces.

Under wet conditions, the metal surface was covered with a microscope coverslip and incubated in a humid chamber for the specified contact times (1 h or 24 h) at 20 ± 2 °C in the dark. Under dry conditions, the sample was allowed to dry completely in a biosafety cabinet under aseptic conditions for five minutes. The dry metal surface with the virus was then incubated for the specified contact times (1 h or 24 h) at approximately 50% relative humidity, 20 ± 2 °C, in the dark (Figure 1).

The virus control was incubated in a separate well of a sterile 6-well plastic plate under the same conditions and was analyzed before the experiment and after 1 and 24 h of incubation.

After the contact period, 490 µL of DMEM/F12 medium containing 2% FBS was added to both wet and dry samples. The virus was eluted for 5 min, after which the residual virus was collected by pipetting from the metal surface. Viral samples were immediately titrated to determine TCID_50_ and were also used for DNA extraction. For PCR, aliquots were frozen and stored at –80 °C until analysis.

The experiment was performed twice independently.

#### 2.4.2. Determination of Virus Load and Titre

The viral load was determined for the virus control prior to the experiment and after 1 and 24 h of incubation, and for the virus exposed to the test surfaces after 1 and 24 h of contact.

BoHV-1 titres were evaluated using the 50% tissue culture infectious dose (TCID_50_) method with MDBK cells cultured in 96-well plates (TPP, Switzerland). Viral samples were serially diluted from 10^−1^ to 10^−8^, and cells were infected with the resulting concentrations. DMEM/F12 medium supplemented with 2% FBS was used for titration. From each dilution, 100 µL of the sample was added to four wells. Infected cells were monitored microscopically at 24 h intervals for cytopathic effects, with the final assessment performed after 5 days.

The virus titre and limit of detection (LoD) were calculated by the Spearman-Kerber method using Marco Binder’s TCID_50_ calculator [34]. The virus titre was recorded as the mean in decimal logarithms (log_10_), along with the standard deviation (±SD) [35].

The log_10_ reduction factor of metals was evaluated as described by Ruppach. Virus reduction potential was classified as high (≥4 log_10_), moderate (≥ 2 and <4 log_10_), indicative (≥1 and <2 log_10_), or low (<1 log_10_) according to the log_10_ reduction factor [33].

#### 2.4.3. Determination of Changes in the Quantity of BoHV-1 DNA

Quantitative PCR (TaqMan) was performed to detect BoHV-1 DNA. Quantitative changes in BoHV-1 DNA were evaluated based on variations in Ct values depending on the type of metal surface.

DNA was isolated using the Genomic DNA Purification Kit (Thermo Fisher Scientific, Waltham, MA, USA; K0721) following the procedure described by Lelešius et al. [36].

Quantitative PCR was performed in accordance with the method described by Abril et al. [37]. Primers and probes for qPCR were designed using Primer Express software (version 1.0; Applied Biosystems, Foster City, CA, USA) to amplify the BoHV-1 glycoprotein gB gene sequence (nt 2106–2202) within the open reading frame (ORF). The oligonucleotide primers and the MGB (minor groove binder)-labeled probe were synthesized by Invitrogen (Thermo Fisher Scientific, Waltham, MA, USA).

PCR was performed according to the standard protocol. Quantitative PCR reactions were carried out in a total volume of 25 μL, consisting of 12.5 μL of TaqMan Universal Master Mix II (Applied Biosystems, Thermo Fisher Scientific, Waltham, MA, USA; catalog no. 4440038), 1.6 μL of the primer–probe mixture, and 10.9 μL of the DNA sample, which was prepared by diluting 2 μL of the original sample with 8.9 μL of nuclease-free water. The final concentrations of primers and probe were as follows: BoHV-1 forward primer, 240 nM; BoHV-1 reverse primer, 240 nM; and BoHV-1 probe, 160 nM (Table 2).

The PCR cycling conditions were as follows: initial incubation at 50 °C for 2 min and denaturation at 95 °C for 10 min, followed by 40 cycles of denaturation at 95 °C for 15 s and annealing/extension at 60 °C for 1 min. Reactions were performed using a Mastercycler RealPlex^2^ thermocycler (Eppendorf AG, Hamburg, Germany). Quantitative PCR threshold cycle (Ct) values and standard deviations were calculated. For the qPCR assessment, a series of 10-fold DNA dilutions was prepared to generate a standard curve. The R^2^ value and slope of this curve were evaluated to confirm the linearity and efficiency of the reaction.

#### 2.4.4. Calculation of Virus and Viral DNA Half-Life (t_1/2_)

The virus and viral DNA inactivation time was calculated using the formula [38]:

$$N\left(t\right)={N_{0}\left(\frac{1}{2}\right)}^{\frac{t}{t_{1/2}}}\;⟹\;t_{1/2}=\frac{-ln\left(2\right)\cdot t}{ln\left(\frac{N\left(t\right)}{N_{0}}\right)}$$

Explanations: *N(t)*—virus number or *Ct* value after contact, *N_0_*—initial virus number or *Ct* value, *t*—incubation and contact time, *t_1/2_*—half-life.

T_1/2_ was calculated according to Gracely et al.’s recommendations [39], i.e., the formula used the absolute recalculated virus number, not the virus titres expressed in logarithms. The half-life of the viral DNA was calculated for two intervals: after 1 h and after 24 h. The half-life of viral DNA was calculated by comparing Ct values before contact and after 1 h (for the first hour), and then by comparing Ct values after 1 h and 24 h (for the subsequent 23 h).

### 2.5. Statistical Analysis

The effectiveness of metals was compared after 1 and 24 h of exposure to the virus under wet and dry conditions by evaluating the dynamics of virus inactivation (virus reduction factor), as well as qPCR Ct values and BoHV-1 DNA half-lives under the same conditions (metals, contact time, wet and dry conditions). Differences between groups were assessed using Fisher’s exact test and Student’s t-test, and the Pearson correlation coefficient (R) was also calculated. Differences were considered statistically significant at *p* < 0.05. Statistical analysis was performed using Microsoft Excel 2013 and IBM^®^ SPSS^®^ Statistics (IBM Corp., Armonk, NY, USA), version 29.

## 3. Results

### 3.1. Cytotoxicity Control

Cytotoxicity controls of metal surfaces were performed to distinguish cytotoxic from non-cytotoxic concentrations for MDBK cells. Copper surfaces were found to be cytotoxic at a dilution factor of 1:25, where cell viability was reduced by more than 50%, whereas at a dilution of 1:50, cell viability remained above 70%, indicating no cytotoxic effect at this concentration. In contrast, zinc and steel surfaces were non-cytotoxic. Importantly, cytotoxicity did not affect the outcomes of the virucidal assays, as cytopathic effects (CPE) were observed at non-cytotoxic concentrations.

### 3.2. Effects of Metal Surfaces on BoHV-1

**Figure 2:**
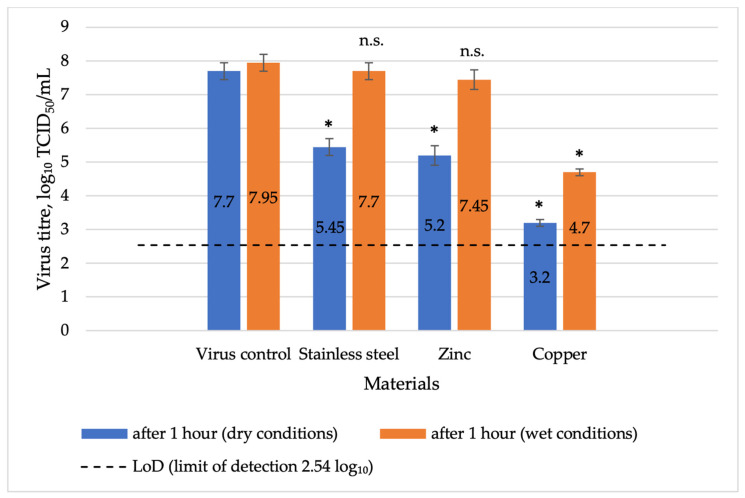
Comparative virucidal effect of various metals after 1 h of exposure to herpesvirus under wet and dry conditions, including a virus control before the experiment. Explanations: LoD-limit of detection 2.54 log_10_; * *p* < 0.05 compared to virus control, n.s. *p* ≥ 0.05 compared to virus control.

The virucidal effect of metal surfaces on herpesviruses under different conditions was evaluated. After 1 h of contact under dry conditions (Figure 2), copper exhibited a high virus reduction potential (a virus reduction factor of 4.5 log_10_, statistically significant, *p* = 0.002) with the virus titre decreasing to 3.2 log_10_ ± 0.1 TCID_50_/mL. In contrast, zinc and stainless steel surfaces showed moderate reduction potential, with a statistically significant reduction in viral titre after 1 h (*p* = 0.007 and *p* = 0.014 respectively).

After 1 h of contact under wet conditions, copper achieved a statistically significant reduction (*p* = 0.007 and exhibited the highest level of effectiveness, with a moderate virus reduction potential (3.25 log_10_), reducing the virus titre to 4.7 ± 0.29 log_10_ TCID_50_/mL (Figure 2). In contrast, zinc and stainless steel showed statistically insignificant changes (*p* = 0.184 and *p* = 0.453 respectively; Figure 2) and a low virus reduction potential (virus log_10_ reduction up to 0.5 log_10_).

**Figure 3:**
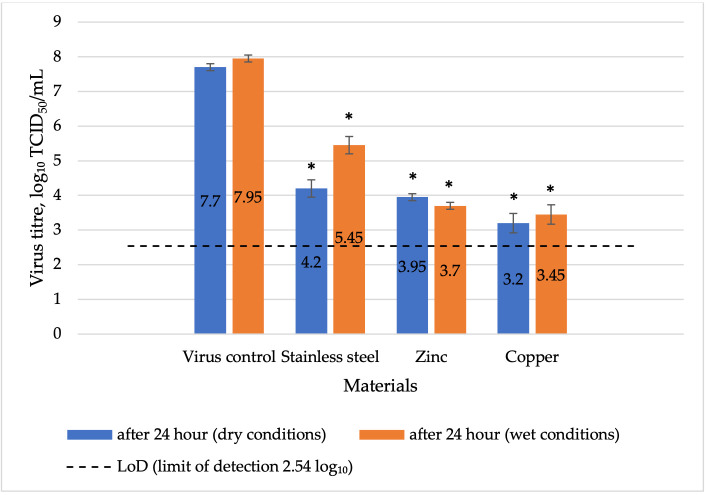
Comparative virucidal effect of various metals after 24 h of exposure to herpesvirus under wet and dry conditions, including a virus control before the experiment. Explanations: LoD-limit of detection 2.54 log_10_; * *p* < 0.05 compared to virus control.

After 24 h of contact between herpesvirus and metal surfaces under dry conditions, a significant reduction in virus titre was observed for all tested materials. Copper exhibited high reduction potential, while zinc and stainless steel exhibited moderate reduction potential. After contact with copper, the virus titre decreased to 3.2 ± 0.1 log_10_ TCID_50_/mL, with a virus reduction factor of 4.5 log_10_ (*p* = 0.002; Figure 3), while on the zinc-coated surface, the virus titre decreased to 4.2 ± 0.1 log_10_ TCID_50_/mL, with a virus reduction factor of 3.5 log_10_ (*p* = 0.004), and on stainless steel, the titre reached 3.95 ± 0.25 log_10_ TCID_50_/mL, corresponding to a reduction factor of 3.75 log_10_ (*p* = 0.005).

Under wet conditions, a significant reduction in virus titre was observed after 24 h of contact with all tested metal surfaces. Copper and zinc exhibited high virus reduction potential, while stainless steel showed moderate potential. Copper exhibited the strongest effect, with a virus titre of 3.45 log_10_ ± 0.25 TCID_50_/mL and a reduction factor of 4.5 log_10_ (*p* = 0.003; Figure 3). Zinc decreased the virus titre to 3.7± 0.28 log_10_ mL TCID_50_/mL, corresponding to a reduction factor of 4.25 log_10_ (*p* = 0.004), while stainless steel decreased the virus titre to 5.45 ± 0.25 log_10_ TCID_50_/mL, corresponding to a reduction factor of 2.5 log_10_ (*p* = 0.01).

### 3.3. Assessment of Virus Titres and qPCR Ct Values After Exposure to Metal Surfaces

After performing qPCR, the Ct values of samples exposed to the metal surfaces for different durations and under wet and dry conditions were compared (Table 3). The data presented in the table show that contact with copper resulted in the greatest increase in Ct values.

It was also found that after contact with different metal surfaces, the virus titre exhibited a negative correlation with qPCR Ct values. The Pearson correlation coefficient (R), which measures the strength and direction of a linear relationship between two variables, was calculated. After 1 h of contact under wet conditions, the correlation coefficient was R = −0.8913, and under dry conditions after 1 h it was R = −0.9815. Under wet conditions after 24 h, the correlation coefficient was R = −0.8548, while under dry conditions after 24 h it reached R = −0.9991. These results indicate a strong negative correlation between BoHV-1 titre and Ct values across all tested conditions.

### 3.4. BoHV-1 and DNA Half-Life upon Contact with Various Metal Surfaces

#### 3.4.1. BoHV-1 Half-Life upon Contact with Various Metal Surfaces

**Figure 4:**
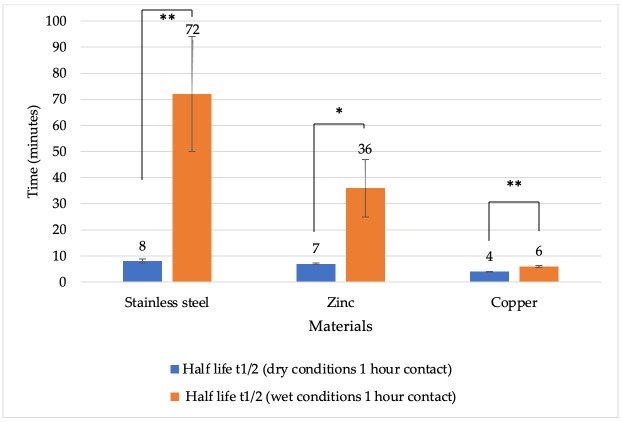
Half-life of BoHV-1 (minutes) on various metal surfaces under dry and wet conditions after 1 h of contact. Explanations: * *p* < 0.05, ** *p* < 0.01.

To evaluate the effect of metal surfaces on BoHV-1 under different conditions, the rates of viral inactivation were assessed by calculating the viral half-life as described by Gracely et al. [39]. After 1 h of contact under dry conditions, the half-life was shorter on all metal surfaces compared to wet conditions (Figure 4). The shortest viral half-life was recorded on copper surfaces under both dry and wet conditions. Specifically, the half-life of copper was 4 min under dry conditions and 6 min under wet conditions. On zinc, the viral half-life was 7 min under dry conditions and 36 min under wet conditions. For stainless steel, the half-life was 8 min under dry conditions and 72 min under wet conditions. A statistically significant difference was observed after 1 h under both dry and wet conditions between stainless steel and copper (*p* = 0.0008 and *p* = 0.0065, respectively), as well as between zinc and copper (*p* = 0.0001 and *p* = 0.0092, respectively).

**Figure 5:**
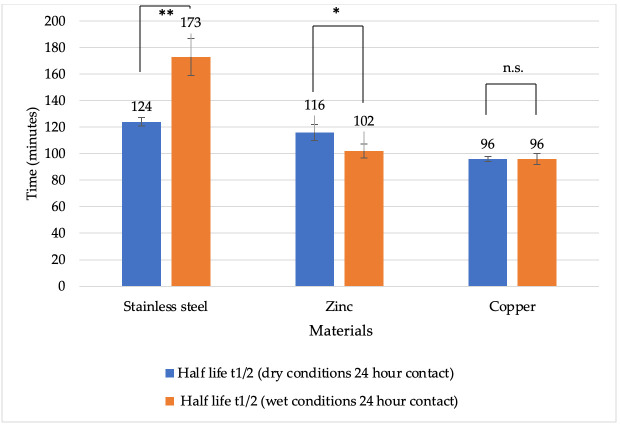
Half-life of BoHV-1 (minutes) on different metal surfaces under dry and wet conditions after 24 h of contact. Explanations: * *p* < 0.05, ** *p* < 0.01, n.s. *p* ≥ 0.05 (not significant).

The virucidal effect of different metals was compared after 24 h of exposure. Overall, the viral half-life was shorter under wet conditions (Figure 5). On copper, the half-life after 24 h of contact was up to 96 min under dry conditions and 96 min under wet conditions. On zinc surfaces, the half-life was 116 min under dry conditions and 102 min under wet conditions. For stainless steel, the half-life was 124 min under dry conditions and 173 min under wet conditions. A statistically significant difference was observed after 24 h under dry conditions between stainless steel and copper (*p* = 0.0002), as well as between zinc and copper (*p* = 0.0055). In contrast, after 24 h under wet conditions, statistically significant differences were observed between stainless steel and zinc (*p* = 0.0012), as well as between stainless steel and copper (*p* = 0.0008).

It was also found that, at both 1 and 24 h, under dry and wet conditions, the virus half-life was shortest on the copper surface (Figure 4 and Figure 5).

#### 3.4.2. BoHV-1 DNA Half-Life Time in Contact with Different Metal Surfaces

The qPCR analysis demonstrated a standard curve with an R^2^ value of 0.991 and a slope of −3.37, corresponding to a reaction efficiency of approximately 98.6%**,** indicating high linearity and highly efficient amplification. Based on these results, Ct measurements were used to assess the decrease in DNA quantity across the dilution series, with increasing Ct values reflecting progressively lower template concentrations. These Ct shifts were subsequently applied to calculate the half-life of the target DNA, without any correction for reaction efficiency.

**Figure 6:**
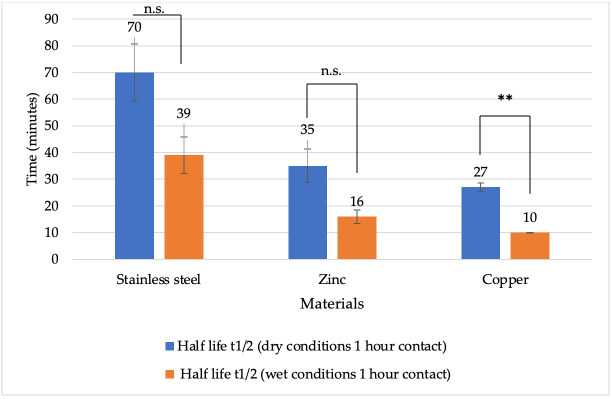
Half-life of BoHV-1 DNA (minutes) on different metal surfaces under dry and wet conditions after 1 h of contact. Explanations: ** *p* < 0.01, n.s. *p* ≥ 0.05 (not significant).

In this study, the effect of metal surfaces on BoHV-1 DNA under different conditions was assessed by analysing viral DNA half-life times. After one hour of contact, viral DNA half-life was generally longer under dry conditions compared to wet conditions (Figure 6). The shortest DNA half-life was observed on copper surfaces under both dry and wet conditions, with 27 min under dry conditions and 10 min under wet conditions. On zinc surfaces, viral DNA half-life after 1 h of contact was 35 min under dry conditions and 16 min under wet conditions. On stainless steel, the corresponding half-life times were 1 h 10 min under dry conditions and 38 min under wet conditions. A statistically significant difference after 1 h under dry conditions was observed between stainless steel and copper (*p* = 0.0297), whereas under wet conditions, significant differences were found between stainless steel and zinc (*p* = 0.0471), as well as between stainless steel and copper (*p* = 0.0268).

**Figure 7:**
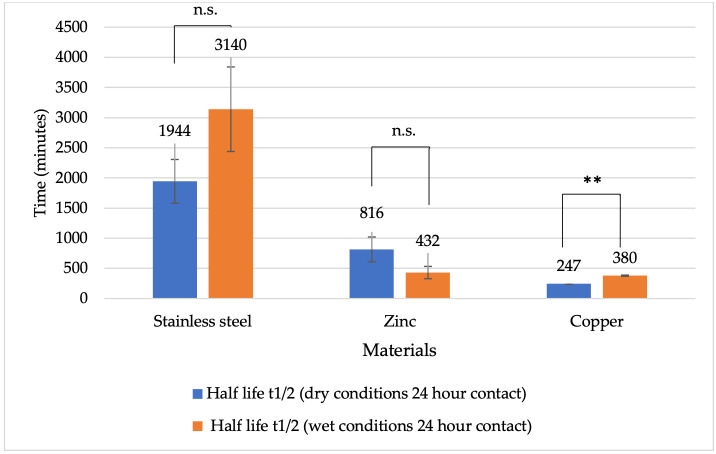
Half-life of BoHV-1 DNA (minutes) on different metal surfaces under dry and wet conditions after 24 h of contact. Explanations: ** *p* < 0.01, n.s. *p* ≥ 0.05 (not significant).

A comparison of the virucidal effects of different metals over 1 and 24 h periods showed that viral DNA half-life was generally shorter under dry conditions (Figure 7). On stainless steel surfaces, viral DNA half-life after 24 h of contact was 32 h 24 min under dry conditions and 52 h 20 min under wet conditions. For zinc surfaces, viral DNA half-life after 24 h of contact was longer under dry conditions (13 h 36 min) than under wet conditions (7 h 12 min). On copper surfaces, the mean viral DNA half-life after 24 h of contact was 4 h 7 min under dry conditions and 6 h 20 min under wet conditions. Overall, for both 1 and 24 h periods and under both dry and wet conditions, the greatest reduction in viral DNA half-life occurred when viruses were in contact with copper surfaces (Figure 6 and Figure 7). After 24 h under dry conditions, a statistically significant difference was observed only between stainless steel and copper (*p* = 0.0221), whereas under wet conditions, significant differences were found between stainless steel and zinc (*p* = 0.0326), as well as between stainless steel and copper (*p* = 0.0308).

## 4. Discussion

Surfaces play an important role in the transmission of infectious diseases. However, most studies examining the antiviral properties of metals and their alloys have focused primarily on human, monkey, or murine viruses [40,41], and considerably less is known about animal pathogens. The animal pathogen BoHV-1 investigated in this study is closely related to HSV-1, a virus of major importance in human medicine and the most extensively studied representative of the *Orthoherpesviridae* family. As a consequence, individuals infected with herpesviruses become lifelong carriers and may intermittently shed varying amounts of virus over shorter or longer periods throughout their lives [42]. Despite their similarities, BoHV-1 also exhibits differences that may influence its susceptibility to chemical, physical, or biological factors in the environment. In particular, variations in L-particle production, tegument composition, and envelopment indicate that alphaherpesvirus morphogenesis is not uniform across species and may affect the stability and infectivity of viral particles under environmental conditions [43].

The metals—copper, zinc, and stainless steel—were selected for our study because they are commonly used in virucidal research, and many products in animal environments are made of or can be coated with these materials [42,44]. We investigated the effects of these metal surfaces on BoHV-1 infectivity and stability to evaluate their potential virucidal activity under both wet and dry conditions. The results showed that the amount of BoHV-1 decreased on all tested surfaces at different rates, although complete (100%) inactivation was not achieved. In practice, viruses present in different, either stable or changing, environments come into contact with surfaces composed of various materials, which may be either dry or wet, and can remain on them for varying periods of time. Even if not all viruses are inactivated, reducing their quantity can still be significant if the remaining amount is insufficient to reach an effective infectious or lethal dose. Both rapid virus inactivation over short contact times and strong virucidal effects during prolonged exposure are important, particularly when high viral loads must be reduced to minimal residual levels.

In our study, when evaluating surfaces of copper, zinc, and stainless steel, we observed that after 1 h of contact under both wet and dry conditions, the copper surface exhibited the strongest virucidal effect. It reduced BoHV-1 titres to 4.7 log_10_ and 3.2 log_10_ TCID_50_/mL, corresponding to 99.9% and 99.9968% inactivation of BoHV-1, respectively. The zinc surface showed a weaker virucidal effect than copper but a stronger effect than stainless steel under both wet and dry conditions within one hour. Accordingly, 64.5% (wet conditions) and 99.683% (after drying) of the virus were inactivated on the zinc surface, compared to 43.8% and 99.44% on stainless steel.

Comparisons between dry and wet conditions indicated a greater virucidal effect under dry conditions. This may be explained by the drying process itself, which can damage enveloped viruses such as BoHV-1. Dry conditions promote direct contact between virus particles and the metal surface, enhancing susceptibility to metal-mediated inactivation. Additionally, liquid evaporation can concentrate metal ions on the surface, facilitating faster interaction with viral particles and accelerating inactivation [45].

Recently, surfaces coated with metal nanolayers, as an alternative to metal alloys, have attracted considerable attention [46,47]. In a study investigating the effect of CuO nanoparticles (CuO-NPs) on HSV-1, a non-cytotoxic copper concentration of 100 μg/mL reduced viral titres by 2.8 log_10_ TCID_50_, demonstrating that their virucidal efficacy can be comparable to that of metal alloys. Several factors have been identified as contributing to the antiviral activity of CuO, including the generation of ROS through free copper ions released from the nanoparticles, which can oxidize viral proteins or degrade the viral genome, thereby inactivating HSV-1 [48].

The virucidal action of metals is based on several mechanisms, including rapid restructuring of viral surface proteins and membranes, damage to the viral envelope, increased permeability of the capsid and plasma membrane, and direct destruction of external viral structures. Dissolved metal ions can induce membrane depolarization, ultimately preventing viruses from attaching to and entering target cells. Viral genomic nucleic acids exposed to copper showed non-specific degradation and fragmentation, with the extent of damage increasing with longer exposure times [46,49,50].

Zinc is considered a potentially effective surface material for preventing the spread of infectious diseases. Studies have shown that zinc exhibits antiviral activity and possesses antiviral effects, against human herpesviruses [51,52]. The results of our study showed that zinc inactivated more than 99.99% of BoHV-1 under wet conditions and more than 99.98% under dry conditions after 24 h, indicating that prolonged contact between the virus and the surface is required. Zinc has shown antiviral activity against numerous virus species, although the precise mechanisms of its virucidal action remain under investigation [53,54,55,56]. Observed effects of zinc include the inhibition of viral enzymatic activities, such as proteases and RNA/DNA polymerases, as well as interference with physical processes critical for viral infection, including virus attachment to target cells and entry into susceptible cells, potentially through depolarization of viral envelope receptors [57,58]. It is hypothesized that the biocidal effect of zinc is mediated by several factors, including the generation of ROS in the vicinity of zinc particles, disruption of energy-generating cellular systems leading to the breakdown of protective membranes, interference with DNA replication, and the initiation of genomic material degradation [59]. Zinc particles have been observed to bind to virions and interfere with viral DNA polymerase activity, thereby inhibiting viral replication [53,60].

CuO shows moderate antiviral activity. While it is considerably more active than ZnO, both exhibit a measurable virucidal effect. The antiviral properties of CuO against viruses can be attributed to the formation of ROS, as well as specific catalytic activity. During incubation of Cu/Zn nanocoatings with virus-containing droplets, virus particles can be inactivated through direct contact with the metal surface and through interactions with released ions (${\mathrm{Cu}}^{2+}$ and ${\mathrm{Zn}}^{2+}$). An analysis of the transport of virus particles in solution compared to the diffusion of released ions, based on the Stokes–Einstein equation, has shown that virus particle movement is much slower than ion diffusion. This indicates that ionic antiviral effects occur more rapidly than contact-based inactivation [56].

Our study demonstrated that after 24 h of contact under both dry and wet conditions, a significant proportion of viruses were inactivated on all tested metal surfaces, with copper showing the highest virucidal effectiveness. The amounts of virus remaining on zinc-coated and stainless-steel surfaces after 1 and 24 h of contact were not statistically different from each other. In contrast, virus titres on the copper surface were significantly reduced (*p* < 0.05).

Quantitative PCR analysis of BoHV-1 after contact with metal surfaces under various conditions revealed a negative correlation between virus titre and Ct values. Ct values increase when viral DNA is degraded, reflecting a decrease in virus titre. Notably, after 24 h of contact under wet conditions, zinc also demonstrated substantial antiviral activity, with a virus reduction factor comparable to that of copper. While wet surfaces generally support longer virus survival, prolonged contact of the liquid with the metal allows metal ions or metal compounds to enter the medium, thereby more effectively targeting viral surface proteins and nucleic acids [61].

Determination of virus half-life times (t½) showed that, after one hour of contact with all metal surfaces, virus titre decreased most rapidly in dried samples under dry conditions. During this initial hour, t½ ranged from 4 to 8 min across all surfaces. When dry samples were incubated for 24 h, the median time required to inactivate 50% of the virus population increased from 96 min on copper surfaces to over 277 min on zinc and stainless-steel surfaces. This effect can be attributed to the increased concentrations of copper and zinc ions in the virus-containing medium during drying. At the same time, the reduced sample volume facilitated more efficient direct contact between virus particles and the metal surface, resulting in a greater number of affected viruses [45]. Over a longer period (up to 24 h), virus titre declined more rapidly under wet conditions.

Stainless steel is the most commonly used reference surface in virucidal studies comparing different metals and their alloys [1,3,12,13,26,61,62,63]. BoHV-1 inactivation on stainless steel proceeded more slowly, with a weaker effect, and virus titre and Ct values changed relatively little compared to other metals. AISI 304L stainless steel naturally forms a 1–3 nm thick passive oxide layer on its surface, primarily composed of chromium oxide (Cr_2_O_3_) in the inner region and chromium hydroxide (Cr(OH)_3_) in the outer region, with minor amounts of iron oxides (e.g., FeO, Fe_2_O_3_, Fe_3_O_4_). This chromium-enriched layer acts as a protective barrier, making the material highly resistant to general corrosion and relatively inert in many environments [64].

The exact mechanism of the virus inactivation process is not fully understood; however, studies suggest that factors such as increased concentrations of sodium and potassium ions, loss of structural water from the surface, disruption of hydrophobic bonds due to water evaporation, and pH changes under oxygen exposure play a major role in virus inactivation during medium drying [51,65,66].

It is hypothesized that virus inactivation occurs more rapidly during the initial phase of incubation (the first hour). This phenomenon resembles biphasic half-life (elimination) kinetics, which has also been observed in pharmacokinetic studies of drugs [67]. However, reliable demonstration of biphasic inactivation requires at least five measurement time points, as also applied in previous studies [68,69,70]. In the context of the present study, the BoHV-1 population appeared to be heterogeneous, with the most sensitive viruses inactivated first, while the more resistant viruses persisted, slowing the overall inactivation process and increasing the half-life (t½). Consistent with these kinetic observations, particularly in the case of herpesviruses, PCR remains a highly sensitive tool for detecting viral DNA; however, it cannot distinguish between infectious and inactivated particles. For evaluating the virucidal efficacy of materials, live virus assays are mandatory. Research using these methods confirms that herpesvirus DNA persists significantly longer than its infectivity, proving that viral degradation is a multi-stage process and highlighting the importance of viability testing in environmental monitoring [71].

## 5. Conclusions

In summary, the results indicate that herpesviruses were inactivated most rapidly on copper-coated surfaces. Zinc also exhibited a virucidal effect, although the inactivation was slower and required prolonged contact. The kinetics of herpesvirus inactivation were influenced by the environmental conditions: in wet samples, virus inactivation proceeded more slowly, whereas in dried samples, inactivation occurred rapidly within the first hour of exposure. However, when considering a 24 h contact period, the viral half-life increased significantly, particularly under dry conditions. Future studies could be conducted using more frequent sampling time points (e.g., 30 min, 3, 6, 12, 24, and 48 h, etc.), as well as apply similar methodologies to more resistant, non-enveloped viruses such as rotaviruses and parvoviruses.

## Figures and Tables

**Table 1:** Metals used in the experiment and their specification.

Metal	Specification
Copper	Grade 11000, contains 99.9% pure copper [27].
Zinc	Galvanised steel, grade DX51D + Z275 [28].
Iron	Stainless steel, grade AISI 304L (1.4307) [29].

**Table 2:** Primers and probe used for qPCR detection of the BoHV-1 gB locus [37].

Oligo-Nucleotides	Sequence (5′-3′)	Product Length (bp)
BoHV-1 forward	TGT GGA CCT AAA CCT CAC GGT	
BoHV-1 reverse	GTA GTC GAG CAG ACC CGT GTC	97
BoHV-1 probe ^a^	AGG ACC GCG AGT TCT TGC CGC	

^a^–MGB (Minor groove binder).

**Table 3:** Assessment of the impact of metal surfaces on BoHV-1 DNA levels by qPCR. Explanations: Mean Ct ± SD–cycle threshold ± standard deviation; * the value of virus control before experiment.

	qPCR, Mean Ct ± SD			
	Conditions			
	After 1 h		After 24 h	
	Wet	Dry	Wet	Dry
Stainless steel	31.67 ± 0.33	30.98 ± 0.16	31.23 ± 0.44	31.69 ± 0.16
Zinc	33.87 ± 0.73	31.80 ± 0.89	37.07 ± 0.83	33.49 ± 0.90
Copper	36.02 ± 0.08	32.33 ± 0.14	39.69 ± 0.15	37.92 ± 0.25
Virus control *	30.12 ± 0.06	30.12 ± 0.06	30.12 ± 0.06	30.12 ± 0.06

## Data Availability

The datasets supporting the results of this document are contained within the article. Any additional data may be requested to the corresponding author.

## Abbreviations

The following abbreviations are used in this manuscript:

BoHV-1	Bovine herpesvirus type 1
BoHV-2	Bovine herpesvirus type 2
BoHV-4	Bovine herpesvirus type 4
BoHV-5	Bovine herpesvirus type 5
CPE	Cytopathic effect
Ct	Threshold cycle
CuO	Copper oxide
DMEM/F12	Dulbecco’s Modified Eagle Medium and Nutrient Mixture F-12
DNA	Deoxyribonucleic acid
FBS	Fetal bovine serum
HSV-1	Herpes simplex virus type 1
MDBK	Madin–Darby bovine embryonic kidney
MGB	Minor groove binder
NPs	Nanoparticles
PCR	Polymerase chain reaction
RNA	Ribonucleic acid
ROS	Reactive oxygen species
